# Dosimetric assessment of prostate cancer patients through principal component analysis (PCA)

**DOI:** 10.1120/jacmp.v14i1.3882

**Published:** 2013-01-07

**Authors:** Aime M Gloi, Robert Buchanan

**Affiliations:** ^1^ Department of Radiation Oncology St Vincent Hospital Green Bay WI; ^2^ Department of Radiation Oncology Southeast Alabama Medical Center Dothan AL USA

**Keywords:** EUD, exact logistic regression, NTCP, principal component analysis, TCP

## Abstract

The aims of this study were twofold: first, to determine the impact of variance in dose‐volume histograms (DVH) on patient‐specific toxicity after 2 high‐dose fractions in a sample of 22 men with prostate cancer; and second, to compare the effectiveness of traditional DVH analysis and principal component analysis (PCA) in predicting rectum and urethra toxicity. A series of 22 patients diagnosed with prostate adenocarcinoma was treated with 45 Gy external beam and 20 Gy dose rate brachytherapy. Principal component analysis was applied to model the shapes of the rectum and urethra dose‐volume histograms. We used logistic regression to measure the correlations between the principal components and the incidence of rectal bleeding and urethra stricture. We also calculated the equivalent uniform dose (EUD) and normal tissue complication probability (NTCP) for the urethra and rectum, and tumor control probability (TCP) for the prostate using BioSuite software. We evaluated their correlations with rectal and urethra toxicity. The rectum DVHs are well described by one principal component (PC1), which accounts for 93.5% of the variance in their shapes. The urethra DVHs are described by two principal components, PC1 and PC2, which account for 94.98% and 3.15% of the variance, respectively. Multivariate exact logistic regression suggests that urethra PC2 is a good predictor of stricture, with Nagelkerke's $R^{2}$ estimated at 0.798 and a Wald criterion of 5.421 (p<0.021). The average NTCPs were 0.06%±0.04% and 1.25%±0.22% for the rectum and urethra, respectively. The average TCP was 85.29%±2.28%. This study suggests that principal component analysis can be used to identify the shape variation in dose‐volume histograms, and that the principal components can be correlated with the toxicity of a treatment plan based on multivariate analysis. The principal components are also correlated with traditional dosimetric parameters.

PACS number: 3.6.96.0

## I. INTRODUCTION

Adenocarcinoma of the prostate is a slowly growing tumor, with an incidence of 25.3 per 100,000.(1) Its treatment options include laparoscopic or radical prostatectomy, external beam radiation therapy (EBRT), EBRT with high‐dose rate (HDR) delivered by remote afterloading brachytherapy, HDR remote afterloading brachytherapy alone, and permanent source interstitial low‐dose brachytherapy. Of the present treatment options, prostate brachytherapy has become widespread because it delivers a lower dose to organs in the vicinity, while giving the maximum dose to the prostate. The modalities with higher dose rates have been associated with better tumor control.

The prevalence of three‐dimensional (3D) models in treatment planning, namely volumetric datasets and dose‐volume histograms (DVHs), has been important in quantifying and predicting treatment outcomes. A DVH allows for the computation of essential biological parameters such as tumor control probability (TCP) and normal tissue complication probability (NTCP). Several models based on DVHs^(^ ^2^ ^–^ ^5^ ^)^ have been used to evaluate patient treatment plans and design clinical protocols. However, researchers are using different methods and parameters to construct their DVHs.^(^ ^2^ ^,^ ^3^ ^,^ ^6^ ^–^ ^9^ ^)^ For example, one stream of literature debates the merits of correlating a single DVH with the maximum dose or the use‐equivalent uniform dose (EUD)^(^ ^10^ ^,^ ^11^ ^)^ with toxicity.^(^ ^5^ ^,^ ^12^ ^–^ ^13^ ^)^

For prostate HDR, the main dose‐limiting organs are the bladder, urethra, and rectum. Several studies^(^ ^5^ ^,^ ^7^ ^,^ ^8^ ^,^ ^9^ ^)^ have found significant correlations between parameters derived from DVHs and the incidence of bleeding or urethra stricture. The drawback to this approach is the variability of choices made in treating patients and reducing the DVH to a single parameter for correlation analysis. For example, in a given patient cohort, the beam direction of the treatment technique influences the shape of the treatment area and the relative heights of bins in the DVH. The bins of a DVH are, therefore, always highly correlated with each other. To analyze the correlated variability of a DVH, we apply principal component analysis (PCA). This method estimates the eigenvectors of the covariance matrix between histogram bins, in order to find independent ‘eigenmodes’ of the histogram shape. The corresponding eigenvalues are objective parameters that can be correlated against the side effects of a proposed treatment plan.

Some other reports in the literature describe the use of PCA to quantify complication risk for various organs. Sohn et al.^(^ ^5^ ^)^ use PCA to analyze the variability of DVH shapes in a patient population and correlate the PCA parameters with late bleeding. Dawson et al.^(^ ^12^ ^)^ describe a novel method using PCA to analyze partial volume effects in normal liver and parotid gland tissues exposed to radiation, and correlate the dose distribution with complication risk. Two studies from Bauer et al.^(^ ^13^ ^,^ ^14^ ^)^ concentrate on datasets of 52 and 119 rectal wall DVHs, and found correlations between the dominant principal components and rectal bleeding of Grade 2 or greater. In this article, we present recent experiments scrutinizing the inherent relationships among several parameters related to prostate treatment. In this study, we use the William Beaumont Hospital protocol for locally advanced prostate cancer patients. The protocol consisted of 25 fractions (180 Gy each) EBRT, in combination with conformal 2 fractions (10 Gy each) HDR brachytherapy. This method has been demonstrated to provide good long‐term tumor control with a low risk of distant metastases,^(^ ^15^ ^–^ ^17^ ^)^ lower PSA nadir levels, longer time intervals to PSA nadir, and improved biochemical control compared with treatment with conventional doses of EBRT alone as reported by Kestin et al.^(^ ^18^ ^)^

Admittedly, the small sample size limited the conclusions that could have been drawn from the study model, but we have been comforted by studies done by Mundt et al.^(^ ^19^ ^)^ in which 37 women were used for GI toxicity, and Fawzy et al.^(^ ^20^ ^)^ where 32 patients were being evaluated. A report released by Devisetty et al.^(^ ^21^ ^)^ has a sample of 48 patients with anal cancer treated with concurrent chemotherapy and IMRT at the University of Chicago from October 2000 to June 2006.

Specifically, we apply PCA to the dose‐volume histograms (DVHs) of the patients and derive the coefficients of the most important principal components (PCs). We measure the correlation between the PC components and chronic rectal bleeding of Grade 2 or greater using exact logistic regression analysis. A radiobiological software package, BioSuite, was used to estimate prostate TCP, urethra NTCP, and rectum NTCP.

## II. MATERIALS AND METHODS

### A. Equivalent uniform dose (EUD), tumor control probability (TCP), normal tissue complication probability (NTCP)

EUD, TCP, and NTCP were estimated for each treatment plan using BioSuite, a radiobiological software tool.^(^ ^22^ ^)^ We define EUD, based on the idea originally proposed by Niemierko et al.,^(^ ^10^ ^)^ as the uniformly distributed dose that will lead to the same level of cell killing as a given nonuniform distribution. TCP calculations were derived from a Poisson model. The data are modeled assuming α/β=1.49 Gy and $\alpha =0.0391{\text{Gy}}^{-1}$ (see Fowler et al.^(^ ^23^ ^)^. Wand et al.^(^ ^24^ ^)^ have estimated in previous studies that the number of clonogens for high‐risk patient groups is $1.1\times 10^{7}$.

Rectal NTCPs were estimated using a Lyman‐Kutcher‐Burman (LKB)^(^ ^25^ ^)^ model for rectal bleeding. The endpoints for rectal bleeding were set using α/β=3.0 Gy, the volume effect n=0.085, the slope m=0.27, and ${\text{TD}}_{50}=97.70\;\text{Gy}$.^(^ ^26^ ^)^ The NTCP distributions for the urethra were calculated to estimate urethra shrinkage, ulceration, and stricture. This model uses the parameters α/β=5.0, n=0.085, m=0.27, and ${\text{TD}}_{50}=60\;\text{Gy}$.^(^ ^26^ ^)^

### B. Principal component analysis (PCA) and exact logistic regression

Principal component analysis is an algorithm that reduces the dimensionality of data while retaining most of the variation in the dataset.^(^ ^27^ ^)^ It identifies linearly independent combinations of parameters that summarize the statistical correlations present in the data. PCA is an orthogonal linear transformation; that is, the principal components define a new orthogonal coordinate system. It is defined such that the greatest variance within the data occurs along the first coordinate (the first principal component); the second‐greatest variance occurs along the second coordinate, and so on.^(^ ^27^ ^)^ The first component is the eigenvector with the largest eigenvalue, and represents the most important source of variation in the data. The last component represents the least important process contributing to the variation. In this paper, the correlations between PC coefficients and toxicity are assessed using exact logistic regression. Exact logistic regression is used to model binary outcome variables in which the log odd of the outcome is modeled as a linear combination of the predictor variables. It is used when the sample size is too small for a regular logistic regression (which uses the standard maximum‐likelihood‐based estimator). The dependent variable in each case is a binary state: “0” for bleeding less than Grade 2, “1” for bleeding of Grade 2 or greater, “0” for less urethra stricture, and “1” for equal or greater stricture.

### C. Patients and the treatment technique

We identified a cohort of patients selected for a HDR brachytherapy boost. All of them had high‐grade, localized prostate cancer and were medically fit to receive and benefit from the treatment. They received neoadjuvant androgen deprivation therapy, followed by EBRT to the prostate with 45 Gy delivered in 25 fractions over a period of five weeks. Typically, after the first week of EBRT, a HDR brachytherapy boost to the prostate is administered after an interval of one to two weeks. The HDR brachytherapy boost procedure is as follows. The patients were placed in the lithotomy position, then urologists inserted catheters via a transrectal ultrasound probe using a perineal template to help guide the needles. Two gold fiducial markers (one at the base and one at the apex; Alpha‐Omega Services, Bellflower, CA) and an average of 17 stainless steel needle catheters (18 gauge, 20 cm long; Varian Medical Systems, Palo Alto, CA) were then inserted into the prostate. The needle positions were adjusted based on a sagittal ultrasound view. The patient was then transferred to a CT scanner (LightSpeed VCTXTe, Miwaukee, WI), where 3 mm slice images of the prostate were obtained. The images were transferred to the treatment plan software (BrachyVision 8.1 and Eclipse; Varian Oncology Systems, Palo Alto, CA) for contouring. The rectum, urethra, and bladder were identified, and the clinical target volume (CTV) for treatment was selected. Finally, based on the CTV, doses were calculated and dwell times were adjusted to optimize the treatment plan. The goals are to cover the CTV with the 100% isodose with a margin of 3 mm, to limit the urethra dose to a maximum of 115% of the prescribed dose (10 Gy), and to limit the rectum dose to between 75% and 80% of the prescribed dose.

## III. RESULTS

Table 1 shows the results of the Kaiser‐Meyer‐Olkin (KMO) test and Barlett's test for the rectum and urethra. These two tests indicate the sampling adequacy and the common variance due to underlying factors, respectively. The analysis was carried out using the Statistical Package for Social Sciences (IBM SPSS 19, 2011). The KMO results are 0.965 and 0.934 for the rectum and urethra, respectively. Compared to a standard threshold of 0.5, they confirm the adequacy of the sample and the suitability of PCA. Therefore, we conducted a principal component analysis for the urethra and rectum datasets. PC1 refers to the coefficient of the first principle component, which is associated with the largest variance in the whole dataset. PC2 is the coefficient of the next largest principal component, and so on. The majority of the variance between the patients' DVHs (Figs. 1(a)), 1(b)) can be described using only one or two PCs. For example, in the urethra DVHs, the first PC explains 94.98% of the variance, while the first and second PCs combined explain 98.13% of the variance.

**Table 1:** Results from Kaiser‐Meyer‐Olkin test.

*KMO and Bartlett's Test*		*Rectum*	*Urethra*
Kaiser‐Meyer‐Olkin Measure of Sampling Adequacy		0.965	0.934
Bartlett's Test of Sphericity	approx. Chi‐Square	458663.348	461550.117
	df	990	990
	Sig	0.000	0.000

**Figure 1(a):**
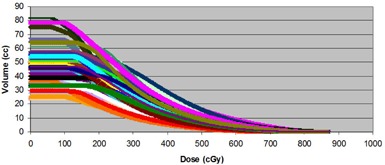
Rectum dose‐volume histograms of all 22 patients treated with 2 fractions of 10 Gy with HDR (boost).

**Figure 1(b):**
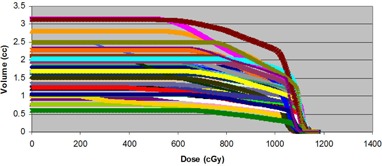
Urethra dose‐volume histograms of all 22 patients treated with 2 fractions of 10 Gy with HDR (boost).

### A. Rectum

About 93.5% of the variability in rectum DVHs is described by the first principal component (PC1). Figure 2(a) illustrates the dominance of PC1 by plotting the amount of variation in the dataset explained by each principal component (a Scree plot). Figure 2(b) illustrates the shape of the first principal component. To group patients into patterns of similar behavior, we subject their scores to a hierarchical, agglomerative cluster analysis (CA) followed by a K‐Means Cluster Analysis (KMCA). The dendrogram derived (Fig. 3(a)) provides a visual summary of the clustering process by linking the closest patients into pairs and smaller clusters.

**Figure 2(a):**
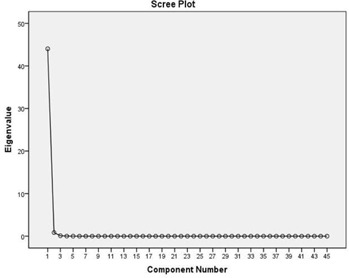
Scree plot indicating the percentage of the variation of rectum DVH described by PC1.

**Figure 2(b):**
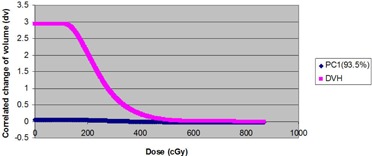
Normed PC1 eigenvector resulting from component analysis of 44 rectum DVHs.

**Figure 3(a):**
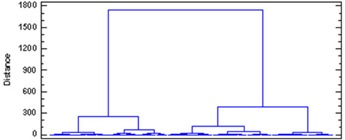
Dendrogram of rectum DVH described by clusters.

### B. Urethra

The urethra DVHs of all 22 patients are illustrated in Fig. 1(b). In this case, we decided to retain the first two principal components. This choice is justified by a Scree plot of the variance explained by each principal component (Fig. 3(b)). Together, the first two PCs describe 98.13% of the variation in the dataset. The shapes of the first two PCs are shown in Fig. 4(a). Plots of PC1 and PC2 (Fig. 4(b)) on a Cartesian plane for both datasets reveal the same clusters of PC values illustrated in Fig. 5.

**Figure 3(b):**
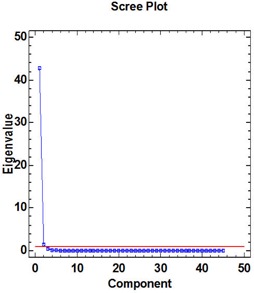
Scree plot indicating the percentage of the variation of rectum DVH described by PC1 and PC2.

**Figure 4(a):**
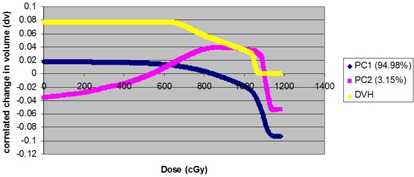
Normed first two eigenvectors resulting from component analysis of 44 urethra DVHs. Percentage of total variance of dataset is described.

**Figure 4(b):**
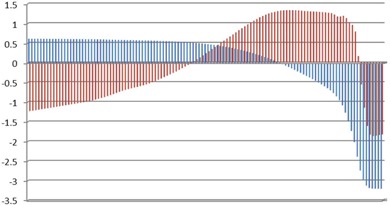
PC1 vs. PC2 for 22 single urethra dose‐volume histograms (PC1 in red and PC2 in blue).

**Figure 5:**
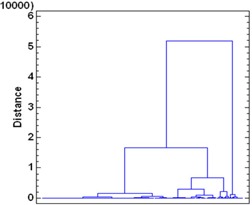
Dendrogram of urethra DVH described several clusters.

### C. Exact logistic regression

Exact logistic regression is used to look for correlations between the coefficients of PC1 and PC2, as well as the PSA test and the Gleason Score. PC1 was the only factor significantly associated with a complication developing in the rectum, whereas PC2 was the main factor in the urethra. There was poor correlation between the PCs and the mean dose for both urethra and rectum DVHs. Moreover, a test of the full model against a constant risk model was statistically significant, indicating that the four predictors distinguish between cases at risk for less or more rectal bleeding and urethra stricture ($X^{2}=492.034$, p<0.000 with df=2 for PC1 rectum; $X^{2}=217.6$, p<0.000 with df=1 for PC2 urethra; $X^{2}=278.343$, p<0.000 with df=1 for PC1 urethra). To study the relationship between toxicity and PCs, we estimated Nagelkerke's $R^{2}$ for the three PC coefficients. The significance of Nagelkerke's $R^{2}$ is estimated using the Wald criterion. The results are $R^{2}=0.798$ with a Wald criterion of 5.421 (p<0.021) for PC2 urethra, $R^{2}=0.996$ with a Wald criterion of 1.616 (p<0.204) for PC1 urethra, and finally $R^{2}=1.000$ with a Wald criterion of 0.086 (p<0.769) for PC1 rectum. These results indicate a moderately strong relationship between toxicity and grouping. However, the Wald criterion demonstrates that only PC2 makes a significant contribution to the toxicity (p=0.021). The prediction success rates are 100% for the rectum DVHs and 98.9% for the urethra DVHs. The results are summarized in Table 2 for the urethra, rectum, and prostate.

**Table 2:** Summary statistics of both rectum and urethra using exact logistics regression analysis.

*Variables*	*Model summary for PC1 Rectum*	*Urethra*
‐2 Log likelihood	0.004	1.263
Cox & Snell $R^{2}$	0.432	0.210
Nagelkerke $R^{2}$	1.000	0.996
Wald criteria	1.218 (p<0.769)	1.616 (p<0.204)
	*Model summary for PC2*	
*Variables*		*Urethra*
‐2 Log likelihood		62.007
Cox & Snell $R^{2}$		0.168
Nagelkerke $R^{2}$		0.798
Wald criteria		5.421(p<0.021)

**Figure 6:**
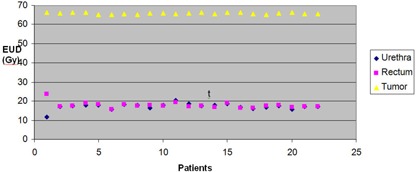
EUD of 22 patients treated with 2 fractions (10 Gy) of HDR (boost).

The patients' EUDs for the urethra, rectum, and prostate are given in Fig. 6. The mean percent difference in the EUD for the urethra is 0.068% (p−value=0.871). The mean percent difference in the EUD for the rectum is 0.032% (p−value=0.926). The calculated average NTCP was 0.06%±0.04% and 1.25%±0.22% for rectum and urethra, respectively. The calculated TCP had an average of 85.29%±2.28%. The relation between rectal overlap volume and the NTCP was not obvious (scattered). The same behavior was seen in the urethra. The mean percent difference for the prostate volume is −0.062% (p−value=0.995). The mean percent differences in NTCP (not shown for each patient) are 0.018% (p−value=0.229) and 0.009% (p−value=0.902) for the rectum and urethra, respectively. The mean percent difference in the TCP was −1.468%±1.767% (p−value=0.1012).

## IV. DISCUSSION

The HDR dose that can be delivered to a prostate carcinoma is largely constrained by the doses received by the bladder, rectum, and urethra. In this study, we require that the maximum dose to urethra not exceed 115% of the prescribed dose, and that the maximum rectum dose be less than 80%. At such high doses, the evaluation of normal tissue toxicity, both acute and delayed, is very important. We have used PCA to analyze the variation between patient dose volume histograms (DVHs). Variation between the rectum DVHs was well described by a single principal component (PC1), whereas the urethra DVHs required two components (PC1 and PC2).

Based on multivariate exact logistic regression, we find that only PC2 in the urethra dataset is correlated with urethra stricture. No comparable data have been published for the urethra, and to our knowledge, no other study has quantified the correlations between HDR parameters using PCA. This research, therefore, illustrates the usefulness of principal component analysis for the interpretation of complex datasets, and demonstrates that PCA can be an effective tool in prostate HDR assessment.

The main idea of this study is to create a feasible model to account for toxicity. We calculated the equivalent uniform doses for prostate, rectum, and urethra in 22 patients. The EUDs derived from this study are comparable to those calculated by Li et al.,^(^ ^28^ ^)^ who used several different schemes. For normal tissue, the rectum data were in good agreement with the results of Söhn et al.^(^ ^5^ ^)^ In addition, we calculated the TCP and NTCP. These are two very useful parameters when planning therapies with very high doses, as they summarize the risk associated with HDR treatment. Such parameters can help us determine how much dose should be considered and delivered to optimize tumor control. Even a slight increase in TCP values may contribute to the uniformity and coverage achieved with each patient. In contrast, Wang et al.^(^ ^29^ ^)^ reported that a decrease in TCP translates into a relapse of the free survival rate. As noted by Tiong et al.,^(^ ^30^ ^)^ a significant decrease in the TCP is related to catheter movement, suggesting that a small (3 mm) tolerance for displacement is acceptable and required. The TCP also decreases significantly if the prostate volume is small. This effect is partially due to a larger percentage of the volume lying outside the PTV of a given shift for small targets. In this study, the TCP values are similar to those reported by Wang et al.^(^ ^29^ ^)^ This study shows considerable consistency between the NTCP values for the urethra and rectum. However, the relationship between parameters like TCP and NTCP and patient outcomes warrants further evaluation in bigger trials.

It was also shown in this study that the principal component with the largest eingenvalue, while it explains the majority of the variation between DVHs, does not necessarily correlate with toxicity. This point is demonstrated in the urethra DVHs, where a larger coefficient for PC2 suggests that the patient will experience less urethra stricture from the HDR treatment. In addition, the study could not correlate DVHs or the derived PC coefficients with overall toxicity. However, this negative result may be attributed to the low rate of toxicity in our population, which has a small number of patients (n=22). Furthermore, the possibility that the DVH shape (i.e., a dominant PC of the DVH) correlates with toxicity is contingent on the treatment technique. If the DVH does not correlate with toxicity, then caution is required when using NTCP as a model base. For example, we were not able to match PC1 of the rectum DVH to rectal toxicity. On the contrary, Dawson et al.,^(^ ^12^ ^)^ Liang et al.,^(^ ^31^ ^)^ Sohn et al.,^(^ ^5^ ^)^ and Bauer et al.^(^ ^13^ ^,^ ^14^ ^)^ demonstrated with great success the relationship between PCs and toxicity using PCA.

We have shown that PCA can be used to provide information on the most meaningful parameters describing the whole dataset, that it is useful for data reduction, and that it accurately summarizes the statistical correlations among variables related to prostate HDR.

## V. CONCLUSIONS

In this work, we investigated the relationships between prostate HDR diagnosis and treatment parameters through PCA. Our study showed that HDR would produce the fewest normal tissue complications when used in conjunction with EBRT. The TCP and NTCP can play a vital role in planning and evaluation when delivering very high doses in individual patients.

## ACKNOWLEGMENTS

The BioSuite Software was kindly provided by julien.uzan@clatterbridgecc.nhs.uk.
